# Challenges hindering family involvement in the hospital nursing care of a child with autism

**DOI:** 10.4102/hsag.v30i0.2731

**Published:** 2025-02-28

**Authors:** Neil A. Williams, Dudu G. Sokhela, Thembelihle S.P. Ngxongo

**Affiliations:** 1 Department of Nursing, Faculty of Health Sciences, Durban University of Technology, Durban, South Africa; 2 Department of Health, King Edward VIII Campus of the Kwa-Zulu Natal College of Nursing, Durban, South Africa

**Keywords:** autism, autism spectrum disorder, barriers, challenges, hindering, family involvement, nursing care

## Abstract

**Background:**

Family involvement is crucial in a child’s treatment; however, many professional nurses still neglect to involve families in their child’s care. Studies have indicated that the perceived lack of family involvement in hospitalised children with autism spectrum disorder (ASD) is a problem in many countries. Few studies have been conducted in Africa with none relating to family involvement, highlighting the need for further research.

**Aim:**

To develop in-depth insights into the challenges regarding family involvement in hospital nursing care of children with ASD, in the South African context.

**Setting:**

Paediatric wards at selected private hospitals and family homes in the eThekwini District of KwaZulu-Natal.

**Methods:**

An interpretative phenomenological analysis method was used. A sample of 10 professional nurses and 10 family members was achieved by purposive sampling. Data were collected from participants using semi-structured in-depth interviews.

**Results:**

The following challenges were identified by the participants: the lack of knowledge of nurses regarding ASD, nurses not listening to family, uncaring attitude of nurses, nurses’ lack of time and shortage of nursing staff.

**Conclusion:**

Nurses play a pivotal role in overcoming the challenges to family involvement, in the hospital nursing care, of a child with ASD. Making nurses aware of the challenges will help improve family involvement in hospitals nursing for the child with ASD.

**Contribution:**

This study added to the body of knowledge by identifying the challenges to the involvement of families in the hospital nursing care of a child with ASD.

## Introduction

Autism spectrum disorder (ASD) is characterised by dramatic impairments in communication, social interaction and repetitive patterns of behaviour, resulting from a neuro-developmental disorder of the brain (Pillai, Makhetha & Aldous 2021:125). Globally, the childhood prevalence of ASD has shown an alarming year-on-year increase (Chiarotti & Venerosi 2020:274). The Centers for Disease Control (CDC) in the United States of America (USA) has estimated that one child out of every 68 is diagnosed with ASD (Center for Disease Control 2014:4). This makes ASD the number one neuro-developmental disorder of children worldwide. However, authors such as Ndubaku (2018:26) report a deficit of knowledge about ASD among professional nurses and healthcare workers. These findings are also true for South Africa, as confirmed by a study conducted in KwaZulu-Natal (KZN) where it was revealed that there has been rapid growth in children diagnosed with ASD and that parents of these children expressed that nurses displayed little ASD-related knowledge (Pillai et al. 2021:126). The deficit in knowledge of ASD by professional nurses has led to poor nursing care of children with ASD (Muskat et al. 2015:482; Russell & McCloskey 2016:21; Taghizadeh et al. 2019:927). This poor nursing care and lack of knowledge can be overcome by closely involving parents in their child’s nursing care (Aarthun, Øymar & Akerjordet 2019:51).

## Problem statement

Hospitalisation of children with ASD can be very stressful for the child because of sensory overload, the impaired ability to communicate, disruption of routine, a new unfamiliar environment and the illness for which the child was admitted. These factors can provoke difficult behaviour in the children such as uncontrolled crying, screaming, biting, scratching and other self-injurious behaviour. It is for this reason, that a nurse, who is not knowledgeable, may find it very difficult to nurse a hospitalised child with ASD (Turgoose et al. 2021:2). This lack of knowledge has resulted in poor nursing care of children with ASD in hospitals (Cranley et al. 2022:70; Garrick et al. 2022:2046). Family members are the first people to notice and monitor signs and symptoms of ASD and are the biggest allies of the medical and nursing team who are treating these children.

Morris, Greenblatt and Saini (2019:2374) highlight a significant gap in knowledge and understanding of the nurse-patient relationship for children with ASD in healthcare settings. These authors further assert that nurses may have insights into the barriers and facilitators affecting this relationship, thus indicating a need for research into their perceptions.

In South Africa generally, and in KZN in particular, a gap exists in the literature with regard to the challenges hindering family involvement in the hospital nursing care of a child with ASD.

### Purpose of the study

The study aimed to develop in-depth insights into the challenges hindering family involvement in the hospital nursing care of a child with ASD in the South African context.

### Research objective

The objective of this research was to identify and describe the challenges perceived by professional nurses and families, in the involvement of families in the hospital nursing care of a child with ASD.

## Research methods and design

The research used a qualitative interpretative phenomenological analysis (IPA) design. The IPA research design was chosen for this study because admitting a child with ASD to the hospital is a highly stressful situation for the child, the family and the professional nurses. It is an experience that can only be described by those who have lived through it. Interpretative phenomenological analysis studies the meaning attributed to the lived experience by a participant and the researcher’s interpretation of this meaning, a process called double hermeneutics (Peat, Rodriguez & Smith 2019:9). This double hermeneutic process enabled the researcher to interpret the meaning attributed by families to the lived experience of having their autistic child admitted to a hospital and how that experience can be improved for other families. Likewise, the researcher interpreted the meaning attributed by paediatric professional nurses to the lived experience of nursing a child with ASD. This information was utilised to triangulate the information received from the family.

### Researcher characteristics and reflexivity

In this study, the researcher practised intersubjective reflexivity by acknowledging that the researcher is a nurse and a parent of a child with ASD, but the researcher did not allow this to impede the data collection and analysis process. The researcher did not allow preconceived ideas to interfere with the research process in any way. The researcher did this by practising bracketing and mindfulness to bolster self-awareness, recording any assumptions in the research plan, keeping a reflexive journal and keeping notes during data collection.

### Study setting

The setting was paediatric wards at selected private hospitals and family homes in the eThekwini District of KZN in South Africa. KwaZulu-Natal is one of the nine provinces of South Africa situated on the east coast.

### Study population and sampling

#### Population

This study had two population groups to ensure triangulation of data. The first group comprised of professional nurses who were permanently employed in the hospital and worked in paediatric wards at the selected private hospitals in the eThekwini region of KZN. There are a total of 68 public hospitals in KZN; 11 (16%) of these are in the eThekwini region. Furthermore, there are 30 private hospitals, of which 18 (60%) are in the eThekwini region. The second group comprised of families of children with ASD whose autistic child had been hospitalised in a paediatric ward in the eThekwini region of KZN. The total population for this study was 42 professional nurses and 50 family members.

#### Sampling

A non-probability purposive sampling technique was utilised as the sampling method for the selection of nurse and family participants who met the inclusion and exclusion criteria (Table 1). The sample size was determined by data saturation. Data saturation was achieved when no new information was gained from new interviews. Two more interviews were carried out after saturation was reached for confirmation. Each participant was interviewed only once.

**TABLE 1:** Sampling technique.

Participants	Sampling method	Inclusion criteria	Exclusion criteria	Sample size
Professional Nurses	Purposive sampling	Professional nurse permanently working in the paediatric ward in a hospital in eThekwini Region of KZN History of nursing a child with ASD	Professional nurses who do not want to share their experience nursing a child with ASD Professional nurses who cannot attend an online interview Enrolled nurses and enrolled nursing auxiliaries working in paediatric ward Professional nurses working in adult wards	Achieved when data saturation was reached. In IPA, small sample sizes are used because of indepth data collection. A total of 42 nurses were approached and 10 sampled
Family	Purposive sampling	Adult family member of a child (< 18 years) with ASD. Child admitted to a hospital paediatric ward in eThekwini region of KZN	Family member of a child not previously admitted to a hospital paediatric ward in eThekwini Family member who is unable to attend an online interview for whatever reason	Achieved when data saturation is reached. In IPA, small sample sizes are used because of in-depth data collection. Advert sent out to 50 family members with 10 sampled

KZN, KwaZulu-Natal; ASD, autism spectrum disorder; IPA, interpretative phenomenological analysis.

#### Inclusion and exclusion criteria

Included in the study were professional nurses permanently employed and working in a paediatric ward and having nursed a child with ASD, and family members of children with ASD who have been admitted to hospital.

All other categories of nurses not working in a paediatric ward, and parents or family members of children admitted to the paediatric ward but did not have ASD were excluded. The researcher did not include any nurses or family members known to the researcher.

### Data collection

All semi-structured one-on-one interviews were conducted online by the researcher using Microsoft Teams video call to minimise the coronavirus disease 2019 (COVID-19) risk. Interviews lasted between 30 min and 60 min and were conducted between November 2021 and January 2022. Interviews were audio recorded on Microsoft Teams.

### Data collection tools

Data were gathered using semi-structured interviews. Audio recordings were used to capture participant responses. Semi-structured interview guides were utilised for nurse and family participants, and were designed to facilitate in-depth discussion with participants and achieve the research objectives. The interview guides were assessed for face validity and content validity by the research supervisor and co-supervisor who were experienced in childhood illnesses and are experts in qualitative research. The tool was developed in English because the researcher, who conducted the interviews, and the interviewees were proficient in English. Data collection tools were pilot-tested prior to conducting the research.

### Data collection tool for nurse participants

The interview guides comprised of three sections: Section 1 elicited information such as the date and time of the interview, the type of employment institution and the participant code. Section 2 gathered demographic information such as marital status, gender, level of education, age and years of service. Section 3 comprised of six open-ended questions that were developed by the researcher based on the research objectives and the literature (Table 2). The researcher used probing questions, when required, to gather more data from participants.

**TABLE 2:** Questions from the interview guide for nurse participants.

Questions
Tell me everything you know about ASD Have you nursed a child with ASD? If yes, describe that experience What is the most important aspect when nursing a child with ASD? What is the role of the family when nursing a child with ASD? What do you believe prevents you from involving family in the nursing care of their child with ASD? What is the best way to involve family in the care of their child in hospital?

ASD, autism spectrum disorder.

### Data collection tool for family participants

This interview guide also comprised of three sections. Section 1 elicited information such as the date and time of the interview and the participant code. Section 2 gathered demographic information such as marital status, age, sex, age of the autistic child and relationship to the child. Section 3 comprised of seven open-ended questions that were developed by the researcher to realise the research objectives (Table 3). The researcher also used probing questions, when required, to gather more data from participants.

**TABLE 3:** Questions from the interview guide for family participants.

Questions
Tell me about your child with ASD When was your child admitted to the hospital? Why was your child admitted to the hospital? What was your perception of the nursing care your child received from the nurses? Explain how you were involved in your child’s care in the hospital? How did you expect the nurse to involve you in the care of your child in the hospital? What do you think prevented you from being more involved, in the nursing care of your child, in the hospital? What was the best way to involve you and your spouse (if applicable) in the in-hospital nursing care of your child?

ASD, autism spectrum disorder.

### Data analysis

Data were analysed after each interview utilising the step-by-step approach of IPA analysis developed by Peat et al. (2019:8). Steps in the IPA process included: (1) reading and intense analysis of the first transcript, (2) as the researcher reads the transcripts, he or she makes observational notes in the margins, (3) the researcher then takes these observational notes and forms groups of data and themes start to emerge, (4) the researcher looks at groups of data and tries to determine how they relate or connect to each other, (5) the researcher then moves on to the next transcript, ‘bracketing’ the themes that emerged in the previous transcript; Steps 1–4 are repeated for each transcript, (6) once all the transcripts have been analysed, common themes across all the transcripts are highlighted and (7) the researcher then looks at the themes across all the transcripts and makes a deeper interpretation to find the meaning of these experiences. Finally, the researcher looks at existing literature to further interpret and analyse the themes.

Transcripts were downloaded and saved in Nvivo (version 12) pro software after each interview. The researcher read individual transcripts line-by-line and listened to the audio tape several times, and then manually coded the material. The researcher coded the transcripts after each interview and then identified themes using an iterative process. The researcher followed the above-described process until data saturation was reached. The themes were validated by two research supervisors who are experts in qualitative and nursing research.

### Ethical considerations

To ensure that this study adhered to all ethical principles, it was reviewed and approved by the Institutional Research Ethics Committee of the Durban University of Technology (Ethical Clearance number: IREC 221/21), the KZN Department of Health (NHRD Ref: KZ_202110_025) and the research/ethics committees of selected private hospitals (Approval number: UNIV-2021-0058). The study adhered to ethical principles of beneficence, respect for human dignity, justice, veracity, and privacy and confidentiality. Participants were informed that they could withdraw at any time. The researcher disclosed the nature of the study, the participant’s right to decline participation and the associated risks and benefits. Participants were interviewed online to minimise the risk of COVID-19. Those who agreed to participate read and signed an information letter and consent form that was emailed to them, and they returned the signed consent via email. Participants’ privacy was protected by using participant codes instead of their names and all collected data were kept under lock and key.

## Results

### Demographic data

The research found that the family participants were married mothers between 28 years and 60 years of age with children 2 – 18 years of age. All nurse participants were female, between 30 and 60 years of age, held a diploma in nursing and had 8 – 30 years of nursing experience. Table 4 and Table 5 detail the demographic information of participants, both family and nurses, respectively.

**TABLE 4:** Demographics of the family participants (*N* = 10).

Participant	Age (years)	Gender	Race/ethnicity	Marital status	Relationship to child	Age of child (years)	Employed outside home	Admitting diagnosis	How long ago was the admission
1	38	Female	African	Married	Mother	4	No	Risperdal overdose	2 years
2	28	Female	African	Single	Mother	16	Yes	Laceration of right hand	2 years
3	29	Female	Indian	Married	Mother	6	No	MRI and audiology test	1 year
4	60	Female	White	Married	Mother	18	No	Dehydration	3 years
5	40	Female	Indian	Married	Mother	2	No	Chest infection	8 months
6	41	female	African	Divorced	Mother	18	Yes	Aggressive behaviour	4 years
7	50	Female	Indian	Married	Mother	16	Yes	Incision and drainage of abscess	2 years
8	30	Female	Mixed race	Married	Mother	7	No	Seizures	1 month
9	51	Female	Indian	Married	Mother	17	Yes	Depression	2 years
10	45	Female	Mixed race	Married	Mother	12	Yes	Dental procedure	2 years

MRI, magnetic resonance imaging.

**TABLE 5:** Demographics of nurse participants (*N* = 10).

Participant	Age (years)	Gender	Race or ethnicity	Marital status	Do you have children	Level of education	Years of service
1	53	Female	Indian	Widowed	Yes	Diploma in general nursing	31
2	50	Female	Indian	Married	Yes	Diploma in general nursing	11
3	50	Female	African	Married	Yes	Diploma in general nursing	30
4	31	Male	Indian	Single	No	Bachelor’s degree	10
5	33	Female	African	Married	Yes	Diploma	13
6	35	Female	Mixed race	Married	Yes	Diploma	15
7	40	Female	White	Married	Yes	Diploma	10
8	30	Female	Indian	Married	Yes	Diploma	10
9	36	Female	Mixed race	Married	Yes	Diploma	4
10	42	Female	Indian	Married	Yes	Bachelor’s degree	16

### Themes

The analysis of data collected from the nurse and family participants were conducted separately, but yielded the same five major themes namely: (1) the lack of knowledge among nurses, (2) nurses not listening to families, (3) uncaring attitudes of nurses, (4) nurses’ lack of time and (5) absence of nursing.

#### Theme 1: The Lack of knowledge

The number one reason verbalised by most family participants was the lack of knowledge by nurses regarding ASD:

‘The nurses don’t understand kids with autism, they thought my son was a danger to the other children. They kept on calling him naughty. They did not have any knowledge of autism at all and they treated my child as if he was mad. I also think that they are don’t know about autism and they have very bad attitudes towards children with autism.’ (F1, F, 38 years)

‘I don’t think the nurses and security guards are clued up at all about autism. They mix up autism with mental health issues. The nurses lack knowledge of autism and they can’t differentiate between autism and bipolar disorder.’ (F2, F, 28 years)

‘The nurses did not know anything about autism and were not trained in autism. They are not trained on how to handle an autistic child. The nurses definitely lack knowledge.’ (F3, F, 29 years)

When nurse participants were asked: ‘What do you think prevents you from involving family members in the nursing care of their child?’, they agreed with family participants that the lack of knowledge was the main reason that parents were not involved:

‘Nurses lack knowledge on ASD and are therefore intimidated and threatened by parents.’ (N1, F, 53 years)

‘It’s just my personal belief. Nurses do not have enough knowledge, so they actually don’t want to be shown up and to receive any question from any parent, because this question might be kind of a challenge to them.’ (N10, F, 42 years)

From the responses, it was apparent that most participants believed that the nurses’ lack of knowledge of ASD was the major challenge in the involvement of families in the hospital nursing care of a child with ASD.

When exploring the knowledge of nurse participants on ASD, the researcher asked: ‘Tell me everything that you know about autism spectrum disorder.’ All nurse participants unanimously agreed that they had very little knowledge of ASD and had received no training:

‘I have no formal training on autism, I am not really clued up on autism. I only know what I have seen on TV.’ (N1, F, 53 years)

‘I have no formal training on autism, and I have very minimal knowledge of autism.’ (N2, F, 50 years)

‘It’s a condition not a disease, the brain does not work normally … I have never had any training on autism.’ (N5, F, 33 years)

Nurses did not have knowledge of nursing a child with ASD and none of them had ever received training on ASD. Nurse participants relied on parents to assist them with nursing tasks and caring for the child with ASD. Some nurse participants felt intimidated and threatened by parents because the parents knew more about ASD than they did. Nurses often felt that they should be the experts who are teaching the parents and not the other way around.

#### Theme 2: Nurses not listening to family

The second theme identified by family participants was that of nurses not listening to them. Family participants identified this as one of the major challenges in the involvement of families in the nursing care of a child with ASD:

‘Even though I told them that he does not react well to the tablet, and he takes the syrup, they did not listen and gave him the tablet anyway. I told the nurses when they put a drip for my child to cover the drip because he will pull it out. The nurses would not listen to me and he pulled it out and he had to have another drip put in, which was traumatising for him. But the nurses refuse to listen to parents and when parents try to explain to the nurses what to do for the child they are told “Don’t tell me my job!”’ (F1, F, 38 years)

‘When I tried to explain to the nurses what happened and what is wrong with him and about autism they would not listen.’ (F2, F, 28 years.

‘When I spoke to the nurse she said “this is how we do it.” They were not listening to us.’ (F3, F, 29 years)

The researcher found that family participants felt disrespected and upset when nurses did not listen to them. Nurses were not listening to family members because they believed that they were medical experts and knew better than them.

Nurse participants did not identify this challenge; however, they did mention that they believed that they should listen to the family. Nurse participants said the following in this regard:

‘Parents know their child best. They know what their child needs. Listen to the parents.’ (N1, F, 53 years)

‘Allow them to tell us what to do for the child because they have the best knowledge of this child … In my opinion the parents know their child the best and they can help to plan the nursing care; we must listen to them.’ (N2, F, 50 years)

‘Firstly, us nurses must listen to the parents, they will tell you the do’s and don’ts. Let the parent’s guide you. The parents can teach us what works at home with the child. The parents can give us information like the child likes and dislikes so that we can use the likes in the nursing care of the child. The parents can tell us if we can play with the child and some of the child’s behaviour like if he or she screams, bites, injures themselves and let the staff know if they do.’ (N9, F, 36 years)

It is apparent from the above-stated results that most family participants, and some nurse participants, regarded nurses as not listening to parents. This was identified as a challenge in the involvement of families in the hospital nursing care of a child with ASD.

#### Theme 3: Uncaring attitude of nurses

The third challenge identified by family participants was the attitude of nurses. Most of the family participants experienced an uncaring and unhelpful attitude of the nurse towards the family and their child with ASD:

‘When they [*nurses*] used to come, they used to say “Oh we going to that naughty one’s room now” and they’d call all the nurses to hold him down because they would say he’s naughty. He wasn’t naughty he was just afraid of the nurses. They were very disrespectful.’ (F1, F, 38 years)

‘Not good at all. The nurses were not helpful, they were not kind. They are not trained on how to handle an autistic child. They handled my child very roughly. When I spoke to the nurse she said “this is how we do it.”’ (F3, F, 29 years)

Family members felt that the poor attitude of nurses was a challenge regarding their involvement in the hospital nursing care of a child with ASD. This poor attitude negatively affected the overall quality of nursing care provided.

Two of the nurse participants directly identified ‘nurses’ attitude’ as a challenge in the involvement of families. Nurse participants suggested that to overcome this challenge, nurses were believed to need patience and a caring attitude:

‘Nurses’ attitude is also a problem, I find that today’s nurses have less compassion for their patients, and they don’t have patience and time when caring for their patients, I feel nurses today are very task oriented and not caring towards their patients. They don’t have a commitment to their patients.’ (N2, F, 50 years)

‘The nurse must have compassion for the child otherwise it will be very difficult.’ (N3, F, 50 years)

From the preceding quotes, it is clear that these nurse participants agreed with 7 out of 10 family participants who reported that poor nurse attitudes were a barrier to family involvement.

#### Theme 4: Nurses’ lack of time

The next theme identified by family participants was ‘Nurses’ lack of time’. Nurse participants also identified this challenge but they perceived it as increased time needed to nurse a child with ASD. Family participants noted that nurses were busy, short-staffed, impatient and were poor at time management:

‘The nurses are too busy and impatient, they’re always in a rush to do their work. They quickly do their work and then go and sit and gossip.’ (F1, F, 38 years)

‘They are short staffed, there are too many patients and too little staff so they lack time with patients.’ (F2, F, 28 years)

‘The nurses don’t have enough time and therefore they can’t cope.’ (F5, F, 40 years)

Half the family participants experienced nurses not having enough time for their children. This sentiment was shared by nurse participants; however, they phrased it differently saying that nursing a child with ASD requires much more time than nursing a neurotypical child. Nurse participants noted the following:

‘Personally, when I first started nursing, I used to avoid nursing children with ASD because they were difficult to nurse and treating them takes much longer than a normal child. Time management is a big barrier to involving parents because it takes much longer. Nurses are normally short staffed and therefore don’t have that extra time to involve the parents in the care of the child.’ (N2, F, 50 years)

‘These patients are very demanding and takes a lot of the nurse’s time, so you have to be patient. When you are nursing this child, you will need to be patient and give more time and attention.’ (N9, F, 36 years)

‘The other reason nurses don’t involve parents is because with these children it takes time and if the mother is not assertive enough, it will take a lot of your time to give this one child medication, whereas you could have finished a whole room full of children.’ (N10, F, 42 years)

#### Theme 5: Absence of nursing staff

The final challenge identified by some family participants was the shortage of nursing staff:

‘They are short staffed. There are too many patients and too little staff so they lack time with patients.’ (F2, F, 28 years)

‘I think there should be one nurse per child to help the children.’ (F3, F, 29 years)

‘Allocate one nurse to care for special needs children that are admitted.’ (F3, F, 29 years)

‘Nurses are short staffed. My son vomited and I pressed the bell 6 times to call the nurses, but eventually I had to clean it myself.’ (F5, F, 40 years)

Shortage of nursing staff was also corroborated by some nurse participants:

‘Time management is a big barrier to involving parents because it takes much longer. Nurses are normally short staffed and therefore don’t have that extra time to involve the parents in the care of the child.’ (N2, F, 50 years)

‘The one child would be aggressive with other children and take their toys and so the mothers would complain to us, the nurse, about this child and all we could say is he has a medical condition. This was frustrating for us as nurses because we had to take time to deal with the mothers complaining and we were short staffed and had 22 patients to care for.’ (N10, F, 42 years)

It was apparent that nurses perceived involving the family in their child’s care to be time-consuming and it compounded the existing staff shortage.

## Measures of trustworthiness

### Triangulation of data

According to Willis et al. (2021:1), data source triangulation involves collecting data from different sources to ensure the validity of the information. The researcher ensured data source triangulation by collecting data from two sources, namely: professional nurses working in paediatric wards and families of children with ASD admitted to paediatric wards. The researcher also used existing literature to validate the findings of the data analysis. Comparison of research findings with reports in the literature is a method used in qualitative research to ensure the validity of the data. Such a process converges data from different sources.

### Trustworthiness

Trustworthiness refers to the integrity of the researcher, the legitimacy of the results and the methodical precision and appropriateness of the research design and method (Rose & Johnson 2020:434). Trustworthiness in this study was achieved by adhering to the four criteria of trustworthiness as described by Lincon and Gruba (1985:301), namely, credibility, transferability, dependability and conformability.

#### Credibility

Rose and Johnson (2020:434) explain credibility as being confident in the certainty of the research findings. Credibility is accomplished when the research findings are an accurate understanding of the data derived from the participants’ true perceptions. Interviews that were conducted with nurse participants and family participants were recorded, and notes were written on the interview guide verbatim, to ensure the capture of participants’ original views. Probing questions were used to ensure data saturation. These notes and recordings will be kept for a period of 5 years after production of the final report, should they be required for verification purposes.

#### Transferability

Transferability is simply the measure to which the research can be reproduced in different settings with different participants to test if it will yield similar findings. Transferability can be ensured by the researcher providing a detailed description of the research (Rose & Johnson 2020:434). Transferability was ensured by providing a thick description of the entire research process, including the data collection, analysis and context of data collection so that this study can be conducted in other settings, contexts or with other respondents

#### Dependability

Dependability is defined by Rose and Johnson (2020:434) as the longevity of findings. Dependability can be achieved by the participants’ assessment of findings regarding authenticity. The researcher ensured dependability by asking the participants to evaluate the findings prior to submitting the research report to the examiners, to ensure their views have not changed over time. None of the participants changed their views and they agreed with the findings. The data collection tools were also pretested prior to data collection. The entire research process was made transparent to ensure dependability.

#### Conformability

Conformability is defined as the extent to which the research findings and interpretations can be endorsed by other researchers to determine accuracy (Rose & Johnson 2020:434). To ensure conformability, the researcher shared recordings and verbatim field notes with research supervisors to ensure that data had been correctly interpreted.

## Discussion

### Demographic data of parents

Most family participants were between the ages of 38 years and 60 years. All participants were female and the primary caregivers of children with ASD. In most countries, including South Africa, the caregiving role is culturally designated to females, and women are socialised into this role from a young age (Asuquo & Akpan-Idiok 2021). Most participants were married. This is corroborated by Abdulmalek and Benkhaial (2018:176) who assert that married caregivers are more likely to provide care to ASD offspring and tend to be more compliant with medical schedules than divorced and widowed caregivers. Finally, contrary to South African racial and ethnic demographics which are predominantly black (Statistics South Africa 2021:1), few of the participants in this study were black Africans and this is a study limitation.

### Demographics of nurse participants

Six of the 10 nurse participants were aged between 30 years and 39 years. The South African Nursing Council (2022:1) reports that 21% of nurses are in this age group suggesting that nurses of other ages need to be examined. Many participants were also noted to be female which was not unsurprising. According to Health e-News (2021), there are overwhelmingly more female nurses in the country than males; nursing is a female-dominated profession.

### Lack of knowledge

Nurse and family participants alike agreed that the lack of knowledge of ASD by nurses was a challenge in the involvement of families in the hospital nursing care of a child with ASD. This finding has been corroborated in other studies also (Ellias & Shah 2019; Hidiroglu et al. 2018; Kostiukow et al. 2020; Mukhamedshina et al. 2022). Family participants were upset by the misdiagnosis and mistreatment of their children and attributed this to nurses’ lack of knowledge about ASD. Where poor knowledge about a common condition exists, patients tend to have poor interpersonal relationships with nurses. A poor interpersonal relationship with nurses was a barrier to effective and meaningful family involvement in the hospital care of the child with ASD. Nurses’ limited knowledge of ASD led nurses to be reluctant to interact with parents – afraid of displaying a lack of knowledge about ASD. This lack of knowledge has proven to be a barrier to the quality of care and family satisfaction in other studies (Morris et al. 2019; Walsh et al. 2020; Wilson & Peterson 2018).

### Nurses not listening to family

The perception of nurse and family participants confirmed that nurses did not listen to family opinions on the nursing care of the child with ASD. The family did not believe that nurses appreciated or recognised their expertise regarding the child’s care. The result was a failure to engage the family in the nursing care the child received. Benich et al. (2018:40) agreed that parents had the best understanding of how their child with ASD responds to stimuli and unfamiliar conditions. It was clear that the majority of the participants believed that a major barrier to being involved in the child’s care in the hospital was that nurses did not listen to the suggestions of parents regarding care. This challenge was also pointed out by Nygård and Clancy (2018:3180) who stated that nurses do not always provide care in a courteous manner, appreciating parents’ experience and skills.

### Nurses’ attitudes

Both nurse and family participants agreed that the uncaring, unhelpful and impatient attitudes of nurses towards the child and family were barriers to the involvement of families in the hospital nursing care of a child with ASD. The nurse’s poor attitude could be attributed to a lack of compassion, caring and time, as well as being intimidated by family members. This perception is supported by literature. A recent article by Cranley et al. (2022:70) confirmed that the views of nurses towards families can either aid or impede family engagement in treatment. While favourable views about families can lead to improved communication, relationships and results, negative attitudes about the family can result in poor sentiments among family members, such as feeling alienated and less empowered to engage in caring. Understanding the view of the nurse on the relevance of family involvement in nursing care is crucial (Hoplock et al. 2019:392). Education of nurses regarding the importance of family in patient care may foster engagement with families, viewing them as a resource. Furthermore, nurses’ knowledge of psychological theories may help them become more proactive and critically reflective in addressing their own beliefs and attitudes towards family involvement (Hoplock et al. 2019:384).

### Nurses lack of time

All participants noted that nurses generally had a lack of time to effectively nurse the child with ASD and also lacked time to involve the family in the nursing care of their child. This challenge is consistent with the literature where Wilson and Peterson (2018:809) found that physicians spent double the appointment time with the child with ASD as opposed to children with other healthcare needs. This time drain finding likely applies to nurses and other healthcare providers who care for children with ASD as well (Walsh et al. 2020:421).

### Absence of nursing staff

Some participants agreed that a shortage of nursing staff was a challenge in the involvement of families in the hospital nursing care of a child with ASD. Jarrar et al. (2018:469) reported that poor patient care often resulted from nurses who were overworked and unable to spend time with the families of patients while they were performing procedures. Because of this, having sufficient staffing levels in the department may enable nurses to spend more time with patients, thereby improving the quality of care. A shortage of staff should be considered as a challenge to family involvement especially where the child has been diagnosed with ASD. Nurses perceive nursing a child with ASD as difficult and time-consuming, leading to a perception of a shortage of staff or a need for specialised staff. It was apparent that nurses perceived involving the family in the care of their child was time-consuming for the nurse and thus they mentioned the shortage of staff.

## Limitations

The study had several limitations. Firstly, the study utilised an IPA methodology with a small sample size of 20 participants and the findings therefore cannot be generalised. Secondly, some parents may have forgotten some of their experiences, particularly when the child was admitted to the hospital a few years ago. Thirdly, it was unknown how long the child had been hospitalised. Length of hospitalisation time, as well as severity of diagnosis, may have influenced responses. Fourthly, a minority of participants were Black South Africans which is in contrast to the predominant race and ethnicity in the country. Additional study of this racial group is needed. Finally, the findings of this study are limited to one district in South Africa, thus limiting the generalisation of the results to other districts. All the nurses interviewed currently work in private hospitals, and the study needs to be repeated in the public hospital setting.

## Recommendations for future research

Based on the findings of this study, the researcher recommends that nurses working in paediatric wards be made aware of the challenges and be empowered to prevent these challenges by developing a relevant policy and framework. The researchers also recommend that this research be repeated in other provinces of South Africa to see if it yields similar findings, and the same research should be conducted with a larger sample of participants, so that the results can be generalised.

## Conclusion

This study has contributed to the body of knowledge in South Africa regarding the challenges hindering family involvement in the hospital nursing care of a child with ASD and has highlighted the pivotal role that nurses can play in family involvement in the hospital care of these children. Communication of study findings can serve to inform policy and nursing practice regarding the care of a child with ASD. The findings from this study also contributed, in part, to the formation of a framework to promote family involvement in the hospital nursing care of a child with ASD.
